# Dietary Supplementation with *Zanthoxylum bungeanum* Seed Cake and Meal Improves the Productive Performance and Antioxidant Capacity of Laying Hens

**DOI:** 10.3390/ani16111611

**Published:** 2026-05-25

**Authors:** Shanchuan Cao, Hanshu Lin, Xiaocong Li, Xinglai Li, Jianfei Zhao, Jingbo Liu

**Affiliations:** College of Life Sciences and Agri-Forestry, Southwest University of Science and Technology, Mianyang 621010, China

**Keywords:** *Zanthoxylum bungeanum* seed cake meal, gut microbiota, liver metabolomics, laying hens

## Abstract

The objective of this paper was to examine the effects of differing levels of dietary *Zanthoxylum bungeanum* seed cake and meal (ZBS) on the productive performance, egg quality, gut microbiota and liver metabolomics of laying hens. In total, 1280 healthy Lohmann Pink laying hens (age, 36 weeks) were randomly assigned to four treatment groups: control (CON), 1% ZBS, 2% ZBS, or 3% ZBS. The trial lasted for eight weeks. The results indicated that compared to the CON group, the 3% ZBS group had a higher egg production during weeks 5–8 and 1–8 (*p* < 0.05). However, the feed-to-egg ratio and egg breakage rate during weeks 1–8 was lower in the 2% ZBS group and 3% ZBS group compared to the CON group (*p* < 0.05). Adding different levels of ZBS to the diet significantly increased egg production, reduced the feed-to-egg ratio, and reduced the feed intake of weeks 5–8 in laying hens (*p* < 0.05) with both effects exhibiting a linear relationship as the addition level increased (*p* < 0.05). Compared to the CON group, supplementing the diet with 2% or 3% ZBS significantly increased T-AOC levels in the liver and serum, as well as T-SOD enzyme activity, but it significantly decreased the MDA level in the serum (*p* < 0.05). The addition of ZBS has been demonstrated to induce alterations in the microbial composition of the cecum in laying hens. The key metabolic pathways and key metabolites are glycerophospholipid metabolism and magnoflorine, respectively. In conclusion, the supplementation of diets with ZBS has been demonstrated to significantly enhance the production performance and antioxidant capacity in laying hens. The optimal supplementation level was 3% ZBS.

## Simple Summary

The feed utilization of agricultural by-products is beneficial for reducing costs and protecting the environment. The *Zanthoxylum bungeanum* seed cake and meal (ZBS) is rich in protein, fat and minerals, and it has strong potential for use as animal feed. This paper found that adding ZBS to the diet of laying hens could enhance their antioxidant capacity and improve production performance.

## 1. Introduction

The laying hen is of significant economic importance to humans, providing a steady supply of nutrient-rich eggs. This makes it a vital component of animal husbandry. It is estimated that feed represents over 65% of total production costs in the context of poultry production [1]. In the context of the competitive poultry farming industry and the volatility of feed ingredient costs, effective cost control is paramount for ensuring the financial viability of poultry enterprises. It is imperative to enhance the scientific understanding of the utilization of cost-effective, readily available agricultural by-products in poultry feed with the objective of reducing feed expenses [2].

*Zanthoxylum bungeanum* seed cake and meal (ZBS) is the residue that is left over after the pressing of *Zanthoxylum bungeanum* seeds for the extraction of oil. In most cases, ZBS is either discarded or utilized as a fertilizer [3,4]. However, ZBS is abundant in nutrients such as protein, fat, and minerals, thus classifying it as an agricultural by-product that has considerable feed utilization value [5]. In addition, some components of ZBS have been demonstrated to possess a range of biological activities, including antibacterial, antitumor, anti-inflammatory, analgesic and antioxidant properties [2,6]. The recycling of ZBS has the potential to contribute to environmental protection and reduce feed costs. Preliminary studies have indicated that the addition of 5–10% *Zanthoxylum bungeanum* seed meal has a substantial effect on the growth and development of broiler chickens whilst concomitantly enhancing the digestibility and utilization of nutrients [7]. Nevertheless, there are no extant reports concerning the feasibility of using ZBS with regard to laying hens. This paper investigated the use of ZBS in layer feed through 16S rRNA and liver metabolomics technologies, providing foundational data for the rational utilization of ZBS.

## 2. Materials and Methods

### 2.1. Preparation and Nutrient Composition of ZBS

The ZBS process in this paper involved the following steps: Dried *Zanthoxylum bungeanum* seeds were roasted at 120 °C for 10 min after impurity removal. The roasted seeds were then pressed through an oil extractor to separate *Zanthoxylum bungeanum* seed oil. The remaining residue constitutes ZBS. The nutritional components of ZBS are listed in Table 1.

### 2.2. Experimental Design, Diets, and Management

In total, 1280 healthy Lohmann Pink laying hens (age, 36 weeks) were randomly assigned to four treatment groups. Each treatment group was performed in eight replicates with 40 hens per replicate (four hens per cage). It is evident that ten adjacent cages, both above and below, constitute a single repetition. The groups were as follows: control (CON), 1% ZBS, 2% ZBS, or 3% ZBS. Preliminary metabolic trials in our lab have determined the metabolic energy of ZBS to be 7.12 MJ/kg, which is a figure comparable to that of wheat bran (5.9 MJ/kg). Consequently, ZBS was utilized as a substitute for wheat bran in this experiment (Table 2). It is asserted that the nutritional content of the daily diet satisfactorily meets the nutritional requirements of laying hens. The trial period lasted for eight weeks. The hens were granted ad libitum access to feed and were housed in an open-ventilated layer house equipped with A-shaped four-tier stainless-steel cages. During the treatment period, the hens were kept at a constant temperature of 20–22 °C and air humidity of 55–60% under a 16:8 light:dark cycle. The hens were provided with adequate access to drinking water.

### 2.3. Production Performance and Sample Collection

The weight and number of all eggs were recorded on a daily basis in order to calculate egg production (total egg production quantity/total rearing days). The collection of weekly feed intake and egg mass data was undertaken in order to determine the average daily feed intake (ADFI) and the feed-to-egg ratio. Following the end of the experiment, a total of 32 eggs were collected from each treatment group (with four eggs being collected for each replicate) for egg quality analysis. For each replicate, one hen was selected. Then, the serum was obtained by centrifuging the wing vein blood samples at 3000× *g* and stored at −80 °C for biochemical indexes and antioxidant analysis. Birds were euthanized by carbon dioxide after blood samples collection; liver tissue samples were collected and stored in cryogenic tubes at −80 °C for metabolomics and antioxidant analysis. In addition, the caecum was isolated from each hen with the cecal contents being collected into separate sample tubes and stored at −80 °C for analyzing gut microbial composition.

### 2.4. Nutrient Analysis

The dry matter content of ZBS was determined by a drying process in an oven at a temperature of 135 °C for a duration of 2 h, as outlined in the AOAC method 930.15 [8]. The crude protein (CP) concentration was determined in accordance with AOAC method 990.03 using an elemental microanalyzer (CHNS-932; Leco Corporation, St. Joseph, MI, USA). The calcium (Ca) and phosphorus (P) concentrations of the diets and ZBS were determined by inductively-coupled plasma spectroscopy (AOAC method 985.01) following wet-ash sample digestion (AOAC method 975.03). The neutral detergent fiber content of amylase-treated ZBS was determined in accordance with the AOAC method 2002.04. The acid detergent fiber content of ZBS was determined in accordance with AOAC method 973.18.

### 2.5. Egg Quality Analysis

The determination of eggshell color, Haugh units, albumen height, and yolk color was facilitated by the utilization of an automatic egg quality analyzer, which was the EMT-5200 model (Robotmation Co., Ltd., Tokyo, Japan). Following the separation of the albumen, the determination of the yolk weight was conducted by means of an electronic scale. The strength of the eggshell was measured using an Eggshell Strength Tester (ETG-1601A; Robotmation Co., Ltd., Tokyo, Japan). The thickness at the blunt end, middle, and tip of the eggshell was measured utilizing a micrometer and subsequently averaged. The calculation of the egg shape index, which encompasses both the transverse and longitudinal measurements, was performed through the utilization of an electronic Vernier caliper (CR-2032; Mahr GmbH, Göttingen, Germany).

### 2.6. Serum Analysis

The following parameters were measured using a fully automated serum biochemical analyzer (HITACH-7020, Hitachi, Japan): alkaline phosphatase (ALP), aspartate aminotransferase (AST), urea, alanine aminotransferase (ALT), albumin (ALB), cholesterol (CHO), triglycerides (TG), and total protein (TP).

This paper estimated the total antioxidant capability (T-AOC) as well as the activities of superoxide dismutase (SOD), catalase (CAT), glutathione peroxidase (GSH-Px), and malondialdehyde (MDA) in serum and liver. The estimation of these parameters was conducted by the utilization of commercially acquired kits (Nanjing Jiancheng Biology Co., Ltd., Nanjing, China), which was in strict accordance with the protocols as stipulated by the manufacturers of these kits.

### 2.7. Liver Metabolomics Analysis

Six samples were randomly selected from each replicate for analysis. A total of 50 mg of solid sample was added to a 2 mL centrifuge tube, and a 6 mm diameter grinding bead was added to 400 µL of extraction solution (methanol:water = 4:1 (*v*:*v*)) containing 0.02 mg/mL of intimal standard (L-2-chlorophenylalanine), which was used for metabolite extraction. Samples were ground by the Wonbio-96c (Shanghai Wanbo Biotechnology Co., Ltd., Shanghai, China) frozen tissue grinder for 6 min (−10 °C, 50 Hz), which was followed by low-temperature ultrasonic extraction for 30 min (5 °C, 40 kHz). The samples were left at −20 °C for 30 min and then centrifuged for 15 min (4 °C, 13,000× *g*), and the supernatant was transferred to the injection vial for LC-MS/MS analysis.

The LC-MS/MS analysis of the samples was conducted on a SCIEX UPLC-Triple TOF 5600 system equipped with an ACQUITY HSS T3 column (100 mm × 2.1 mm i.d., 1.8 um; Waters, USA) at Majorbio Bio-Pharm Technology Co. Ltd (Shanghai, China). The mobile phases consisted of 0.1% formic acid in water: acetonitrile (95:5, *v*/*v*) (solvent A) and 0.1% formic acid in acetonitrile:isopropanol:water (47.5:47.5, *v*/*v*) (solvent B). The flow rate was 0.40 mL/min, and the column temperature was 40 °C.

### 2.8. Gut Microbiome Analysis

Six samples were randomly selected from each replicate for analysis. The 16S rRNA sequencing was performed with the assistance of Shanghai Meiji Biotechnology Co., Ltd. (Shanghai, China) The following steps were taken during the procedure and process of DNA extraction. Following the extraction of genomic DNA from the samples, the extracted DNA was subjected to visualization via 1% agarose gel electrophoresis. For polymerase chain reaction (PCR) amplification, specific primers were selected for the purpose of PCR amplification of the target gene. Primers commonly used for this purpose target the bacterial 16S rRNA gene. Following amplification, the products were analyzed preliminarily via 2% agarose gel electrophoresis before quantification using the QuantiFluor™-ST Blue Fluorescent Quantification System (Promega, Madison, WI, USA). For library preparation and sequencing, the PCR products were purified and quantified prior to the construction of sequencing libraries. The libraries were then processed on a high-throughput sequencing platform. For data quality control, sequenced paired-end (PE) reads were first assembled based on overlap relationships. Concurrently, the quality of the sequence was subject to rigorous control and filtration. Subsequent to sample differentiation, analysis of operational taxonomic units (OTUs) was conducted, which was followed by species taxonomic analysis. It is possible to analyze various diversity indices based on OTUs. The analysis of OTU clustering results enables the assessment of multiple diversity indices for OTUs in addition to the evaluation of sequencing depth. A statistical analysis of community structure is performed at various taxonomic levels with the relevant taxonomic information being used as a basis for this analysis.

### 2.9. Statistical Analysis

Statistical analyses were performed using SAS 9.30 software (SAS Institute, Cary, NC, USA). The replicate was considered an experimental unit for production performance, egg quality, antioxidant capacity and serum biochemical indexes data, which was n = 8 for each group. A laying hen was considered an experimental unit for metabolomics and microbiota analysis data, which was n = 6 for each group. For production performance, egg quality, antioxidant capacity and serum biochemical indexes data, the data were found to conform to a normal distribution by using the Shapiro–Wilk test, one-way analysis of variance (ANOVA), linear and quadratic regression analysis, and Duncan’s method for multiple comparisons. For data (egg breakage rate, dirty egg rate and abnormal egg rate) that do not follow a normal distribution, the Kruskal–Wallis test was used, and linear and quadratic effects were not analyzed.

Statistical analysis was conducted on the data, which were expressed as the mean and standard error of the mean. Statistically, a *p* < 0.05 was considered to be significant.

The R package “ropls” (Version 1.6.2) was used to perform principal component analysis (PCA), orthogonal least partial squares discriminant analysis (OPLS-DA), and 7-cycle interactive validation evaluating the stability of the model. The metabolites with VP > l, *p* < 0.05 were determined as significantly different metabolites based on the variable importance in the projection (VlP) obtained by the OPLS-DA model and the *p*-value generated by Student’s *t* test. Differential metabolites among two groups were mapped into their biochemical pathways through metabolic enrichment and pathway analysis based on the KEGG database. Python packages “scipy.stats” (Version 1.17.1) was used to perform enrichment analysis to obtain the most relevant biological pathways for experiential treatments.

The differences in intergroup microbial communities were assessed using similarity analysis (ANOSIM) and the multiple reaction replacement procedure (MR-PP) for significance testing. The intergroup species differential analysis employed LEfSe analysis. LEfSe’s multi-level species differential analysis (across multiple taxonomic levels: phylum, class, order, family, genus, species) conducted differential testing at multiple hierarchical levels.

## 3. Results

### 3.1. Production Performance and Egg Quality

Compared to the CON group, the 2% ZBS group had a higher feed intake during weeks 5–8 as well as higher egg production during weeks 1–4 and 1–8 (Table 3, *p* < 0.05). Compared to the CON group, the 3% ZBS group had a higher egg production during weeks 1–4, 5–8 and 1–8 (*p* < 0.05). However, the feed-to-egg ratio and egg breakage rate during weeks 1–8 were lower in the 2% ZBS group and 3% ZBS group compared to the CON group (*p* < 0.05). Adding different levels of ZBS to the diet significantly increased egg production, reduced the feed-to-egg ratio and reduced the feed intake of weeks 5–8 in laying hens (*p* < 0.05) with both effects exhibiting a linear relationship as the addition level increased (*p* < 0.05). Additionally, as the ZBS supplementation level increased, the laying rate exhibited a quadratic change between weeks 1–4 and weeks 1–8 (*p* < 0.05). As shown in Table 4, the addition of ZBS significantly reduced eggshell L* while increasing eggshell weight. Both effects exhibited a linear relationship as the addition rate increased (*p* < 0.05). Compared to the CON group, the 3% ZBS group had a lower eggshell L* (*p* < 0.05). The addition of ZBS had no significant effect on the eggshell a* and eggshell b* egg shape index, eggshell strength, albumen height, yolk color, yolk weight, Haugh unit, yolk weight, eggshell weight, eggshell thickness and egg weight (*p* > 0.05).

### 3.2. Antioxidant Capacity and Serum Biochemical

Compared to the CON group, supplementing the diet with 2% or 3% ZBS significantly increased T-AOC levels in the liver and serum, as well as T-SOD enzyme activity, but it significantly decreased the MDA level in the serum (Table 5, *p* < 0.05). At the same time, it showed a linear relationship with increasing ZBS dosage (*p* < 0.05). Serum albumin levels increased linearly with increasing amounts of ZBS (Table 6, *p* < 0.05). The addition of ZBS to the diet had no significant effect on serum biochemical indexes (*p* > 0.05).

### 3.3. Cecal Microbiota

**Figure 1:**
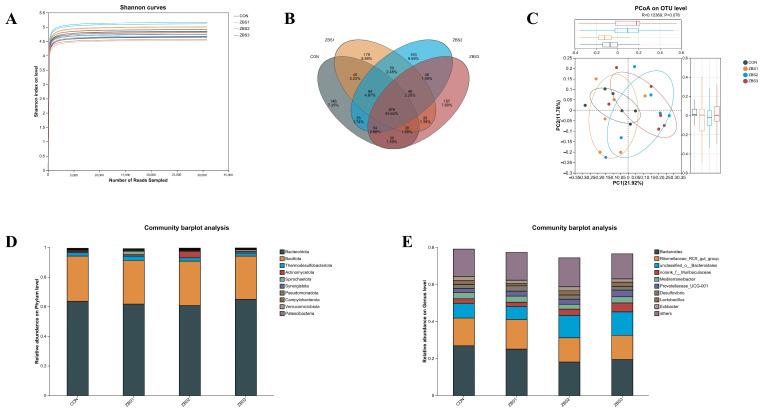
Effects of *Zanthoxylum Bungeanum* seed cake meal supplementation on the microbial composition of the cecum in laying hens. (**A**) Shannon curves. (**B**) Venn graph based on OTU classification. (**C**) PCoA analysis on OUT level. (**D**) Relative abundance on phylum level. (**E**) Relative abundance on genus level.

The Shannon curve flattens toward the end, suggesting that a sufficient amount of sequencing data has been obtained (Figure 1A). The Venn diagram illustrates that each group has its own distinct microbial community (7.25% of CON, 9.89% of ZBS 1, 9.59% of ZBS 2 and 7.8% of ZBS 3, Figure 1B). A PCoA analysis reveals that the OTU composition of each group differs in certain respects (Figure 1C). At the phylum level, the dominant phyla were Bacteroidota, Bacillota, Thermodesulfobacteriota, Actinomycetota, Spirochaetota, Synergistota, Pseudomonadota, Campylobacterota, Verrucomicrobiota and Patescibacteria (Figure 1D). At the genus level, the dominant genus were *Bacteroides*, *Rikenellaceae RC9_gut_group*, *unclassified_o_Bacteroidales*, *norank_f_Muribaculaceae*, *Mediterraneibacter*, *Prevotellaceae_UCG-001*, *Desulfovibrio*, *Lactobacillus* and *Extibacter* (Figure 1E).

**Figure 2:**
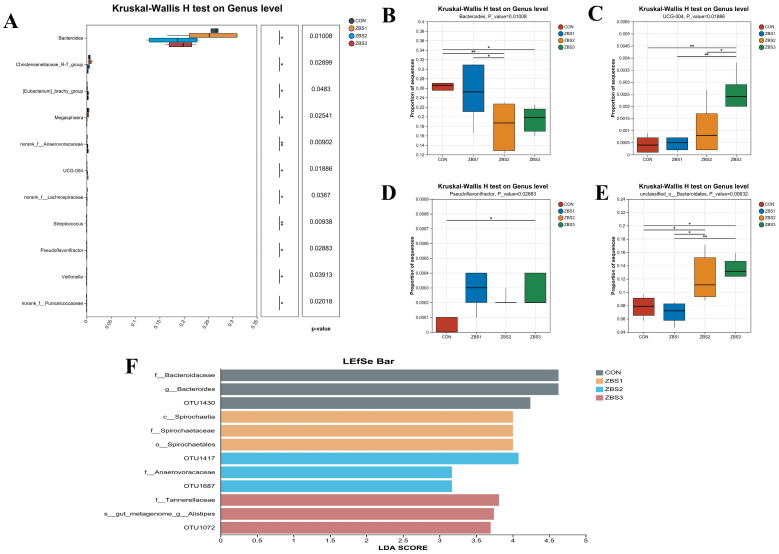
Differential analysis of microbial communities in the cecum of laying hens. (**A**) Kruskal–Wallis H test on Genus’s level (top 10). Kruskal–Wallis H test on (**B**) *Bacteroides*, (**C**) *UCG-004*, (**D**) *Pseudoflavonifractor*, (**E**) *unclassified_o_Bacteroidales*. (**F**) LEfSe multi-level divergence analysis (top 3). * Means *p* < 0.05, ** Means *p* < 0.01.

As shown in Figure 2A, the top 10 genus with significant differences were *Bacteroides*, *Christensenellaceae_R-7_group*, *[Eubacterium]_brachy_group*, *Megasphaera*, *norank_f_Anaerovoracaceae*, *UCG-004*, *norank_f_Lachnospiraceae*, *Streptococcus*, *Pseudoflavonifractor*, *Veillonella,* and *norank_f_Puniceicoccaceae*. Compared to the CON group, the 3% ZBS group had a higher level of *UCG-004*, *Pseudoflavonifractor* and *unclassified_o_Bacteroidales* but a lower level of *Bacteroides* (*p* < 0.05, Figure 2B–E). LEfSe multi-level divergence analysis showed the top three in each group (Figure 2F): f_Bacteroidaceae, g_Bacteroides and OTU1430 for the CON group; c_Spirochaetia, f_Spirochaetaceae and o_Spirochaetales for the 1% ZBS group; OTU1417, f_Anaerovoracaceae and OTU1687 for the 2% ZBS group; and f_Tannerellaceae, s_gut_metagenome_g_Alistipes and OTU1072 for the 3% ZBS group.

### 3.4. Liver Metabolome

**Figure 3:**
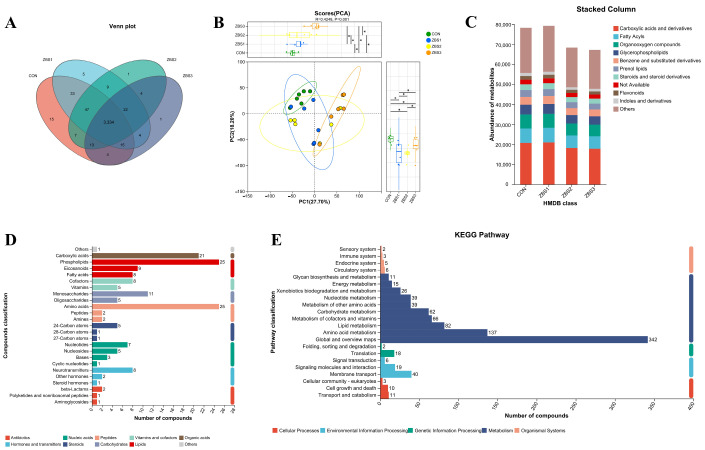
Effects of *Zanthoxylum Bungeanum* seed cake meal supplementation on the liver metabolites in laying hens. (**A**) Venn analysis of liver metabolites. (**B**) PCA analysis. (**C**) Statistical distribution chart of compound classification base on HMDB. (**D**) KEGG compound classification. (**E**) KEGG pathway classification. * Means *p* < 0.05.

Venn diagrams and principal component analysis (PCA) revealed significant differences in the composition of liver metabolites among the groups (*p* < 0.05, Figure 3A,B). After comparing with HMDB, the primary metabolites were identified as carboxylic acids and derivatives, fatty acyls, organooxygen compounds, glycerophospholipids, benzene and substituted derivatives, prenol lipids, steroids and steroid derivatives, flavonoids, indoles and derivatives (Figure 3C). After comparing with KEGG metabolites, the primary metabolites were identified as amino acids, phospholipids and carboxylic acids (Figure 3D). The primary KEGG metabolic pathways involved for all metabolites are as follows: cellular processes, environmental information processing, genetic information processing, metabolism and organismal systems (Figure 3E).

**Figure 4:**
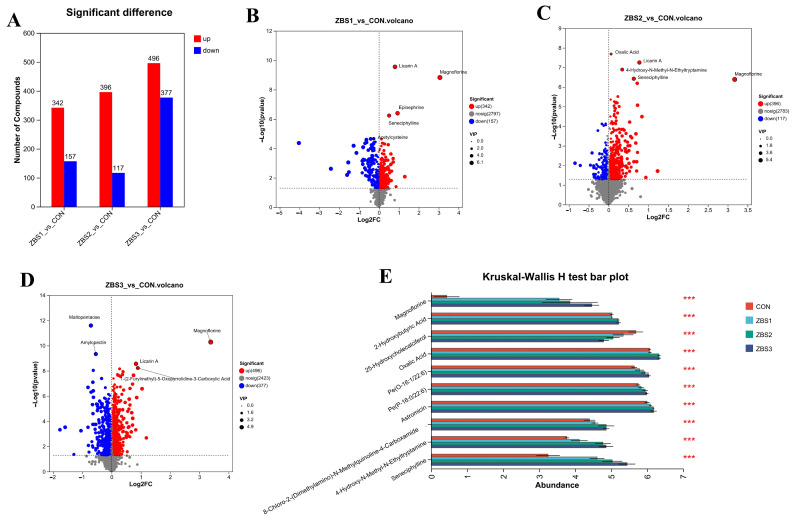
Differential metabolites in laying hen liver. (**A**) Statistics on significant differences. Volcano map of (**B**) ZBS 1 vs. CON, (**C**) ZBS 2 vs. CON and (**D**) ZBS 3 vs. CON. (**E**) Kruskal–Wallis H test bar plot. *** Means *p* < 0.0001.

**Figure 5:**
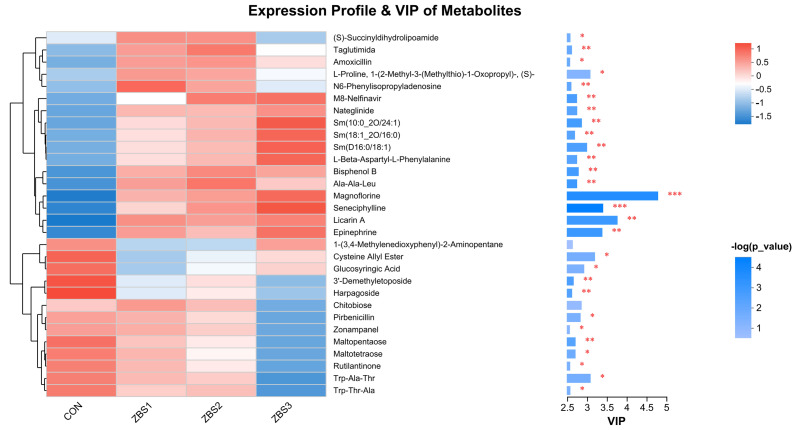
Expression profile and VIP of metabolites in laying hens. * Means *p* < 0.05, ** Means *p* < 0.001, *** Means *p* < 0.0001.

**Figure 6:**
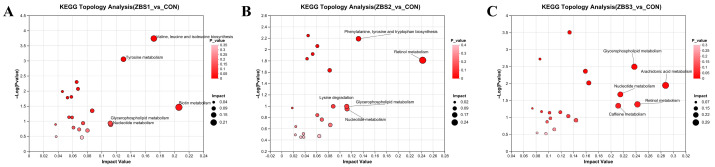
KEGG topology analysis of metabolites in laying hen liver. (**A**) ZBS 1 vs. CON. (**B**) ZBS 2 vs. CON. (**C**) ZBS 3 vs. CON.

Compared with the CON group, the 1% ZBS, 2% ZBS and 3% ZBS groups exhibited an increased expression of 342, 396 and 496 metabolites, respectively, while decreasing the expression of 157, 117 and 377 metabolites (Figure 4A). Figure 3C,D and Figure 4B showed the top five metabolites that differ significantly between the 1% ZBS (licarin a, magnoflorihe, epinephrine, seneciphylline and cetyicysteine), 2% ZBS (oxalic acid, licarin a, 4-hydroxy-n-methyl-n-ethyltryptamine, seneciphylline and magnoflorine) and 3% ZBS (maltopentaose, amylopectin, magnoflorine, licarin a, 1-(2-furylmethyl)-5-oxopyrrolidine-3-carboxylic acid) groups and the CON group, respectively. Figure 4E displays the ten metabolites (magnoflorine, 2-hydroxybutyric acid, 25-hydroxycholecalciferol, oxalic acid, Pe (0–16:1/22:6), Pe (p-16:0/22:6), astromicin, 8-chloro-2-(dimethylamino)-n-methylauinoline-4-carboxamide, 4-hydroxy-n-methy1-n-ethyltryptamine, and seneciphylline) that exhibited the most significant differences between the groups. VIP analysis indicated that magnoflorine was a key metabolite (Figure 5). KEGG topology analysis showed the top five metabolic pathway that differ significantly between the 1% ZBS (valine, leucine and isoleucine biosynthesis, tyrosine metabolism, glycerophospholipid metabolism, nucleotide metabolism, biotin metabolism), 2% ZBS (phenylalanine, tyrosine and tryptophan biosynthesis, retinol metabolism, lysine degradation, glycerophospholipid metabolism, nucleotide metabolism) and 3% ZBS (glycerophospholipid metabolism, arachidonic acid metabolism, nucleotide metabolism, retinol metabolism, caffeine metabolism) groups and the CON group (Figure 6).

## 4. Discussion

The nutritional composition of the ZBS employed in this paper is comparable to that of wheat bran, yet it exhibits a marginally higher nutritional value. This paper investigates the application of ZBS in laying hen diets with the objective of replacing wheat bran. Currently, the market price of ZBS is only about half that of wheat bran. Consequently, utilizing ZBS as a substitute for bran can result in a reduction in feed costs.

Production performance is defined as the economic and efficient capacity of livestock to produce animal products. The findings of this paper demonstrated that the addition of 1%, 2%, and 3% ZBS to the feed of laying hens does not result in any detrimental effects on their production performance. In fact, the results indicated a substantial increase in egg production and decrease in the feed-to-egg ratio. This outcome was entirely unanticipated. We also observed a lower egg breakage rate in the 2% and 3% ZBS groups compared with the CON group. This may be related to their heavier eggshells, although the difference in eggshell strength was not significant during testing. This phenomenon may be ascribed to the polyphenols and flavonoids present in ZBS, which were analogous to those found in *Zanthoxylum bungeanum* [2,6]. During the extraction of *Zanthoxylum bungeanum* seed oil, low-temperature pressing is used to preserve active compounds such as polyphenols and flavonoids [3,4]. A substantial body of research has demonstrated that the supplementation of laying hen diets with plant extracts containing flavonoids and polyphenols has a significant impact on production performance, egg quality, anti-inflammatory capacity, and antioxidant properties [9,10,11,12]. The dietary supplementation of *Zanthoxylum bungeanum* leaf did not result in a reduction in the laying rate [13]. In a similar manner, it was determined that the incorporation of ZBS into the diet resulted in a substantial enhancement of both serum and hepatic antioxidant capacity. In particular, the 3% ZBS group showed significantly increased levels of T-AOC and T-SOD enzyme activity, which reflect the capacity of the antioxidant defense system [14], and significantly reduced MDA content, which is an indicator of oxidative stress [15]. The egg production, feed-to-egg ratio, egg breakage, dirty egg and abnormal egg rate, antioxidant capacity and serum biochemical parameters were similar in the 2% and 3% ZBS groups. However, these increased with dosage. The optimal replacement level is therefore 3%.

The influence of plant polyphenols on the body’s antioxidant capacity was a subject of scientific interest with research demonstrating the potential for these compounds to alter the composition of gut microbiota [16,17]. This paper also investigated the microbial composition of the cecum in laying hens. The results showed that the addition of ZBS led to changes in the gut microbiota. The dominant bacterial phyla were Bacteroidetes and Bacillotes with no significant differences. At the genus level, the abundance of *Bacteroides* in the 2% and 3% ZBS groups were significantly reduced. *Bacteroides*, while involved in nutrient metabolism and immune regulation [18], can also transform into pathogenic bacteria, causing diseases such as enteritis and diarrhea [19]. Additionally, we observed significantly elevated abundances of *UCG-004*, *Pseudoflavonifractor*, and *unclassified_o_Bacteroidales*. The level of *UCG-004* was positively correlated with the fiber content in the diet [20]. The level of *Pseudoflavonifractor* was positively correlated with lipid metabolism [21]. Based on the above results, it is speculated that changes in egg production rates may be attributed to alterations in dietary active components and fiber composition, which in turn lead to changes in nutrient metabolism, particularly lipid metabolism.

With regard to serum biochemistry, no significant differences were observed in the results, indicating that the addition of ZBS did not affect liver function. Additionally, we conducted hepatic metabolomics analysis. The results indicated that 1% ZBS and 2% ZBS share the same differentially expressed metabolites: licarin A and magnqflorine. VIP analysis indicated that magnqflorine has the highest contribution. Magnoflorine represents a significant quaternary aporphine alkaloid that has been isolated from a variety of commonly employed herbal medicines. Magnoflorine has been shown to possess a wide spectrum of pharmacological properties, including anti-diabetic, anti-inflammatory, neuropsychopharmacological, immunomodulatory, hypotensive, antioxidant, and antifungal activities [22,23,24]. It is therefore concluded that magnoflorine was the key metabolite responsible for the increased egg production that was observed when ZBS was added to the diet. Egg yolks contain significant quantities of phospholipids, the synthesis of which is dependent on the glycerophospholipids provided in the diet. It has been demonstrated by preceding studies that abnormalities in the glycerophospholipid metabolic pathway, specifically impaired triglyceride (TAG) synthesis, are the primary cause of reduced egg production [25]. KEGG topology analysis revealed that adding different levels of ZBS ultimately enriched the same metabolic pathways: glycerophospholipid metabolism and nucleotide metabolism. This aligns with the function of the key factor magnoflorine.

It is evident from the results of the microbial and hepatic metabolomics analyses that the addition of ZBS to the diets of laying hens primarily alters production performance by influencing lipid metabolism. The precise mechanisms underlying this effect require further investigation.

## 5. Conclusions

The supplementation of diets with ZBS has been demonstrated to significantly enhance production performance and antioxidant capacity by the regulation of lipid metabolism in laying hens. Magnoflorine has been identified as a significant metabolic factor. The optimal supplementation level was 3% ZBS.

## Figures and Tables

**Table 1:** Nutritional composition of *Zanthoxylum bungeanum* seed cake meal.

Items, %	Content
Dry matter	91.91
ME, MJ/kg	7.12
Crude protein	14.24
Ether extract	5.13
Ash	8.76
ADF	55.42
NDF	61.24
Ca	0.95
P	0.19
Asp	1.33
Thr	0.40
Ser	0.62
Glu	3.05
Gly	0.73
Ala	0.47
Cys	0.26
Val	0.61
Met	0.12
Ile	0.43
Leu	0.87
Tyr	0.41
Phe	0.41
Lys	0.39
His	0.23
Arg	0.55
Pro	0.55
Total amount of 17 amino acids	12.08

**Table 2:** Feed composition and nutritional level (air-dried basis).

Items	*Zanthoxylum bungeanum* Seed Cake Meal			
	0%	1%	2%	3%
Ingredients, g/kg				
Corn	603.0	603.0	603.0	603.0
Soybean meal (43% CP)	240.0	240.0	240.0	240.0
Wheat bran	30.0	20.0	10.0	0.0
Soybean oil	15.0	15.0	15.0	15.0
Limestone	88.0	88.0	88.0	88.0
*Zanthoxylum bungeanum* seed cake meal	0.0	10.0	20.0	30.0
CaHPO_4_	16.0	16.0	16.0	16.0
*L*-Lys•HCl	0.2	0.2	0.2	0.2
*DL*-Met	1.4	1.4	1.4	1.4
*L*-Thr	0.2	0.2	0.2	0.2
NaCl	2.5	2.5	2.5	2.5
NaHCO_3_	1.0	1.0	1.0	1.0
Choline chloride	1.0	1.0	1.0	1.0
Vitamin premix ^(1)^	0.2	0.2	0.2	0.2
Mineral premix ^(2)^	1.5	1.5	1.5	1.5
Total	1000.0	1000.0	1000.0	1000.0
Calculated nutrient levels, %				
ME, MJ/kg	11.22	11.23	11.24	11.26
CP	17.48	17.51	17.54	17.57
Calcium	3.85	3.88	3.92	3.96
Total phosphorus	0.63	0.62	0.62	0.62
Available phosphorus	0.36	0.36	0.36	0.36
Digestible lysine	0.75	0.74	0.76	0.75
Digestible methionine	0.36	0.37	0.38	0.36
Digestible Threonine	0.53	0.54	0.53	0.54
Digestible Tryptophan	0.16	0.17	0.15	0.17

^(1)^ The premix provided the following per kg of diets: CuSO_4_·5H_2_O 8 mg, FeSO_4_·H_2_O 60 mg, MnSO_4_·H_2_O 60 mg, MnSO_4_·H_2_O 80 mg, KI 0.35 mg, Na_2_SeO_3_ 0.30 mg. ^(2)^ The premix provided the following per kg of diets: VA 8000 IU, VB_1_ 0.8 mg, VB_2_ 2.5 mg, D-pantothenic acid 2.2 mg, VB_6_ 3.0 mg, VB_12_ 0.004 mg, VD_3_ 1600 IU, VE 5 IU, VK_3_ 0.5 mg, biotin 0.10 mg, nicotinic acid 20.0 mg.

**Table 3:** Effects of dietary *Zanthoxylum bungeanum* seed cake meal on production performance in laying hens (n = 8).

Items	*Zanthoxylum bungeanum* Seed Cake Meal				SEM	*p*-Value		
	0%	1%	2%	3%		ANOVA	Linear	Quadratic
Feed intake, g/day/hen								
Weeks 1–4	118.23	118.16	118.34	116.64	1.436	0.815	0.482	0.575
Weeks 5–8	113.59 ^b^	115.48 ^ab^	119.54 ^a^	118.16 ^ab^	1.308	0.015	0.005	0.222
Weeks 1–8	116.25	117.01	118.86	116.44	1.320	0.496	0.685	0.238
Egg production, %								
Weeks 1–4	92.56 ^c^	95.01 ^b^	97.47 ^a^	97.32 ^ab^	0.626	0.001	0.001	0.047
Weeks 5–8	90.86 ^b^	92.86 ^ab^	95.91 ^ab^	96.03 ^a^	1.309	0.024	0.004	0.479
Weeks 1–8	90.10 ^b^	94.09 ^a^	96.75 ^a^	96.77 ^a^	0.884	0.001	0.001	0.033
Feed to egg ratio, g/g								
Weeks 1–4	2.15 ^a^	2.07 ^ab^	1.93 ^b^	1.93 ^b^	0.043	0.003	0.001	0.419
Weeks 5–8	2.20 ^a^	2.11 ^ab^	2.06 ^b^	2.03 ^b^	0.036	0.013	0.002	0.369
Weeks 1–8	2.19 ^a^	2.09 ^ab^	1.98 ^b^	1.97 ^b^	0.039	0.001	0.001	0.224
Egg breakage rate, %								
Weeks 1–4	0.88	0.32	0.16	0.23	0.205	0.074	/	/
Weeks 5–8	1.75	0.97	0.32	0.31	0.377	0.035	/	/
Weeks 1–8	1.25 ^a^	0.59 ^ab^	0.23 ^b^	0.27 ^b^	0.218	0.009	/	/
Dirty egg rate, %								
Weeks 1–4	0.16	0.24	0.24	0.23	0.128	0.968	/	/
Weeks 5–8	0.11	0.11	0.40	0.42	0.205	0.552	/	/
Weeks 1–8	0.14	0.19	0.31	0.31	0.142	0.774	/	/
Abnormal egg rate, %								
Weeks 1–4	2.74	1.80	1.47	2.40	0.924	0.762	/	/
Weeks 5–8	1.58	2.00	0.54	1.88	0.731	0.49	/	/
Weeks 1–8	2.29	1.89	1.08	2.18	0.760	0.673	/	/

^a, b, c^ Means in the same row with different superscripts differ significantly (*p* < 0.05).

**Table 4:** Effects of dietary *Zanthoxylum bungeanum* seed cake meal on egg quality in laying hens (n = 8).

Items	*Zanthoxylum bungeanum* Seed Cake Meal				SEM	*p*-Value		
	0%	1%	2%	3%		ANOVA	Linear	Quadratic
Eggshell color								
L*	80.50 ^a^	79.77 ^ab^	79.90 ^a^	77.42 ^b^	0.639	0.009	0.003	0.180
a*	4.96	4.75	4.55	4.22	0.292	0.332	0.070	0.842
b*	13.32	13.56	13.51	13.78	0.503	0.933	0.554	0.975
Egg shape index	1.40	1.39	1.47	1.56	0.098	0.601	0.212	0.628
Eggshell strength, kg/cm^2^	5.50	5.50	5.62	5.59	0.189	0.959	0.660	0.960
Albumen height, mm	6.64	6.78	6.34	6.97	0.261	0.381	0.624	0.348
Yolk color	5.81	5.88	5.73	6.27	0.227	0.351	0.235	0.299
Haugh unit	80.48	82.37	79.35	80.93	1.352	0.473	0.783	0.910
Yolk weight, g	17.34	17.13	17.29	17.85	0.294	0.346	0.205	0.194
Eggshell weight, g	6.83	6.90	6.94	7.23	0.180	0.426	0.133	0.549
Eggshell thickness, mm	0.49	0.47	0.54	0.63	0.099	0.660	0.277	0.554
Egg weight, g	59.88	59.70	61.53	61.19	0.660	0.432	0.374	0.290

^a, b^ Means in the same row with different superscripts differ significantly (*p* < 0.05).

**Table 5:** Effects of dietary *Zanthoxylum bungeanum* seed cake meal on antioxidant capacity in laying hens (n = 8).

Items	*Zanthoxylum bungeanum* Seed Cake Meal				SEM	*p*-Value		
	0%	1%	2%	3%		ANOVA	Linear	Quadratic
Serum								
T-AOC, U/mL	1.84 ^c^	3.26 ^b^	4.63 ^a^	4.92 ^a^	0.268	0.001	0.001	0.045
T-SOD, U/mL	185.30 ^b^	231.59 ^a^	248.80 ^a^	250.07 ^a^	5.057	0.001	0.001	0.001
CAT, U/mL	9.58	10.09	11.07	12.57	1.453	0.493	0.137	0.728
MDA, nmol/mL	6.20 ^a^	5.63 ^ab^	4.19 ^bc^	3.41 ^c^	0.486	0.001	0.001	0.832
Liver								
T-AOC, U/mL	0.28 ^b^	0.45 ^b^	0.92 ^a^	0.97 ^a^	0.114	0.001	0.001	0.572
T-SOD, U/mL	57.89	52.63	59.22	58.27	4.848	0.772	0.724	0.660
CAT, U/mL	11.35	10.17	12.59	13.52	1.397	0.369	0.164	0.458
MDA, nmol/mL	0.14	0.11	0.12	0.13	0.027	0.885	0.755	0.547

^a, b, c^ Means in the same row with different superscripts differ significantly (*p* < 0.05).

**Table 6:** Effects of dietary *Zanthoxylum bungeanum* seed cake meal on serum biochemical indexes in laying hens (n = 8).

Items	*Zanthoxylum bungeanum* Seed Cake Meal				SEM	*p*-Value		
	0%	1%	2%	3%		ANOVA	Linear	Quadratic
Aspartate aminotransferase, U/L	148.03	157.50	151.80	154.51	4.876	0.570	0.533	0.494
UREA, mmol/L	0.69	0.66	0.66	0.63	0.031	0.624	0.217	0.921
Alanine aminotransferase, U/L	7.06	6.41	6.49	6.28	0.847	0.917	0.551	0.798
Albumin, g/L	18.26	20.90	22.12	21.53	1.061	0.075	0.028	0.141
Total cholesterol, mmol/L	1.52	1.57	1.42	1.43	0.089	0.603	0.305	0.845
Triglycerides, mmol/L	5.32	5.19	5.18	4.26	0.491	0.411	0.159	0.425
Total protein, g/L	50.63	51.00	53.00	52.88	1.287	0.442	0.140	0.847

## Data Availability

Data can be made available from the authors upon request.

## References

[1] Thirumalaisamy G., Muralidharan J., Senthilkumar S., Sayee R.H., Priyadharsini M. (2019). Cost-effective feeding of poultry. Int. J. Environ. Sci. Technol..

[2] Nooreen Z., Singh S., Singh D.K., Tandon S., Ahmad A., Luqman S. (2017). Characterization and evaluation of bioactive polyphenolic constituents from *Zanthoxylum Armatum* DC., a Traditionally Used Plant. Biomed. Pharmacother..

[3] Zhang L., Wu H.T., Yang F.X., Zhang J.H. (2015). Evaluation of soxhlet extractor for one-step biodiesel production from *Zanthoxylum Bungeanum* seeds. Fuel Process. Technol..

[4] Bao Y., Yang L., Fu Q., Fu Y., Tian Q., Wang C., Huang Q. (2023). The current situation of *zanthoxylum bungeanum* industry and the research and application prospect. A Review. Fitoterapia.

[5] Li C., Kong Q., Mou H., Jiang Y., Du Y., Zhang F. (2021). Biotransformation of alkylamides and alkaloids by lactic acid bacteria strains isolated from *Zanthoxylum Bungeanum* meal. Bioresour. Technol..

[6] Kumar V., Kumar S., Singh B., Kumar N. (2014). Quantitative and structural analysis of amides and lignans in *Zanthoxylum Armatum* by UPLC-DAD-ESI-QTOF–MS/MS. J. Pharm. Biomed. Anal..

[7] Chen X., Li Y., Zheng A., Wang Z., Wei X., Li S., Purba A., Chen Z., Liu G. (2024). Dietary replacement of soybean meal with *Zanthoxylum bungeanum* seed meal on growth performance, blood parameters, and nutrient utilization in broiler chickens. Animals.

[8] AOAC (2007). Official Methods of Analysis.

[9] Damaziak K., Riedel J., Gozdowski D., Niemiec J., Siennicka A., Róg D. (2017). Productive performance and egg quality of laying hens fed diets supplemented with garlic and onion extracts. J. Appl. Poult. Res..

[10] Dilawar M.A., Mun H.S., Rathnayake D., Yang E.J., Seo Y.S., Park H.S., Yang C.J. (2021). Egg quality parameters, production performance and immunity of laying hens supplemented with plant extracts. Animals.

[11] Abd El-Hack M.E., Salem H.M., Khafaga A.F., Soliman S.M., El-Saadony M.T. (2023). Impacts of polyphenols on laying hens’ productivity and egg quality: A review. J. Anim. Physiol. Anim. Nutr..

[12] Wen K., Zhang K., Gao W., Bai S., Wang J., Song W., Zeng Q., Peng H., Lv L., Xuan Y. (2024). Effects of stevia extract on production performance, serum biochemistry, antioxidant capacity, and gut health of laying hens. Poult. Sci..

[13] Cao S., Lin H., Li X., Zhao J., Lei Q., Zhou J., Wang J., Liu J. (2025). *Zanthoxylum bungeanum* leaf enhanced yolk color and egg flavor in laying hens by altering yolk lipid composition. Poult. Sci..

[14] Wang Y.Z., Xu C.L., An Z.H., Liu J.X., Feng J. (2008). Effect of dietary bovine lactoferrin on performance and antioxidant status of piglets. Anim. Feed Sci. Technol..

[15] Lu T., Piao X.L., Zhang Q., Wang D., Piao X.S., Kim S.W. (2010). Protective effects of *Forsythia suspensa* extract against oxidative stress induced by diquat in rats. Food Chem. Toxicol..

[16] Gavahian M., Mousavi Khaneghah A., Lorenzo J.M., Munekata P.E.S., Garcia-Mantrana I., Collado M.C., Meléndez-Martínez A.J., Barba F.J. (2019). Health benefits of olive oil and its components: Impacts on gut microbiota antioxidant activities, and prevention of noncommunicable diseases. Trends Food Sci. Technol..

[17] Xu X., Guo Y., Chen S., Ma W., Xu X., Hu S., Jin L., Sun J., Mao J., Shen C. (2022). The positive influence of polyphenols extracted from *Pueraria lobata* root on the gut microbiota and its antioxidant capability. Front. Nutr..

[18] Cheng J., Hu J., Geng F., Nie S. (2022). *Bacteroides* utilization for dietary polysaccharides and their beneficial effects on gut health. Food Sci. Hum. Wellness.

[19] Wexler H.M. (2007). Bacteroides: The good, the bad, and the nitty-gritty. Clin. Microbiol. Rev..

[20] An X., Zhang L., Luo J., Zhao S., Jiao T. (2020). Effects of oat hay content in diets on nutrient metabolism and the rumen microflora in sheep. Animals.

[21] Wang Y., Ouyang M., Gao X., Wang S., Fu C., Zeng J., He X. (2020). *Phocea*, *Pseudoflavonifractor* and *Lactobacillus Intestinalis*: Three potential biomarkers of gut microbiota that affect progression and complications of obesity-induced type 2 diabetes mellitus. Diabetes Metab. Syndr. Obes..

[22] Yu S., Yan R., Liang R., Wang W., Yang B. (2012). Bioactive polar compounds from stem bark of *Magnolia Officinalis*. Fitoterapia.

[23] Ahmad W., Jantan I., Kumolosasi E., Haque M.A., Bukhari S.N.A. (2018). Immunomodulatory effects of *Tinospora Crispa* extract and its major compounds on the immune functions of RAW 264.7 macrophages. Int. Immunopharmacol..

[24] Xu T., Kuang T., Du H., Li Q., Feng T., Zhang Y., Fan G. (2020). Magnoflorine: A review of its pharmacology, pharmacokinetics and toxicity. Pharmacol. Res..

[25] Ji Q., Chang P., Dou Y., Zhao Y., Chen X. (2023). Egg yolk fat deposition is regulated by diacylglycerol and ceramide enriched by adipocytokine signaling pathway in laying hens. Animals.

